# Cancer Stem Cells: Notes for Authors

**DOI:** 10.1016/j.stemcr.2020.01.011

**Published:** 2020-02-11

**Authors:** Connie J. Eaves, Martin F. Pera

**Affiliations:** 1 Terry Fox Laboratory, British Columbia Cancer, Vancouver, BC, Canada; 2 The Jackson Laboratory, Bar Harbor, ME, USA; 3 Editor-in-Chief, *Stem Cell Reports*

## Abstract

*Stem Cell Reports* frequently receives manuscripts dealing with the topic of cancer stem cells. Many of the submissions on this topic have major shortcomings in their content or limits to the conclusions that can be drawn from the results presented. The purpose of this Commentary is to highlight some of the underlying issues so that authors can enhance the strength of their research contributions.

## Main Text

*Stem Cell Reports* frequently receives manuscripts dealing with the topic of cancer stem cells. We recognize that this field is an exciting and highly active area of research with potentially profound implications for our understanding of the heterogeneity of malignant cell populations and their genesis, maintenance, response to treatment, and continued diversification. From a translational perspective, the results may also be key to the discovery and testing of new approaches to prognosis and therapy. We therefore welcome submissions that provide new insights into the biology and molecular features of cancer stem cells, as well as new insights into their regulation. Unfortunately, we find that many of the submissions on this topic that we receive have major shortcomings in their content, or limits to the conclusions that can be drawn from the results presented. These contrubtions are therefore returned to authors directly without the delay of a deeper review so that authors can more quickly have the option to submit their work to another journal. The purpose of this Commentary is to highlight some of the underlying issues. Many of these have been covered in detail in an excellent recent review of the cancer stem cell field (Clarke, 2019).

### Limitations of Surrogate Markers to Identify the Behavior of Cancer Stem Cells

We view the concept of a cancer stem cell as a *biological* one. Therefore, use of this term requires some form of rigorous and reproducible experimental measurement of biologically demonstrated stem cell activity. The term “cancer stem cell” was originally introduced to recognize the fact that in most naturally arising cancers, only some of the cells were found to proliferate and only a subset with distinct properties were able to sustain the maintenance of the tumor for an extended period of time. Since current evidence also indicates that many malignant populations contain cells with limited proliferative ability, it is important to distinguish them experimentally from those with a proliferative potential more fitting the definition of cancer stem cells. This has led to the need for measurements that assess cancer cell “stemness” more stringently by demonstrating that a single cell can produce not only a primary population with the properties of clinically accepted features of malignancy but also progeny that include cells that do the same thing. Evidence of this property is now usually reliant on the use of serial transplantation assays *in vivo* or serial organoid formation *in vitro*. However, even these endpoints have shortcomings, as serial transplants are often detecting clones not seen in the primary recipient and hence are not formal evidence of their genesis via a self-renewal division. Expression of a particular cell surface marker or gene expression pattern, or expression of intrinsic cellular or molecular features (e.g., detectability as a side population when labeled with Hoechst 33342, retention of labels that are lost with repetitive cell cycles, or sphere formation *in vitro*), have not yet been shown to isolate any malignant cells with the biological properties of cancer stem cells, as defined above, at sufficient purity to allow them to serve as direct substitute measures of such cells. Data reliant exclusively on such markers or assays have in the past proven to be misleading about cells with the biological properties of cancer stem cells.

At the same time, we recognize that experiments that can be performed in mice or other species can often address properties of cancer stem cells that are difficult or not yet possible to obtain from experiments with human cells because of the inherent limitations of currently available xenotransplantation approaches or *in vitro* systems. For this reason, we welcome studies that make appropriate use of all such model systems.

### Limitations of Established Cancer Cell Lines

Established cell lines have provided many important insights into cancer biology and relevant pathway perturbations that result from specific mutations or epigenetic alterations in cells. However, we feel it is important to recognize that most of the widely available cancer cell lines in use were derived by explanting patient biopsies into simple culture systems that employed basal media supplemented with serum, unmodified tissue culture plastic as a substrate, and dissociation of the original malignant population into single cells with trypsin to select for those able to be continuously passaged and expanded *in vitro* under such conditions. It is now clear that very few primary human malignant cells can survive and proliferate under such circumstances because they display additional physical and molecular requirements for growth *in vitro*. These may include specific niche factors that may be delivered through cell-cell or extracellular matrix contact to support their continued growth *in vivo* or serial propagation. Thus, many of the older, established cell lines are poorly representative of the malignant cell populations from which they originated. Moreover, epigenetic and indeed genetic changes acquired during their adaptation and long-term propagation *in vitro* are likely to have contributed to the acquisition of phenotypes and other properties not relevant to the original malignant cells from which they derived. For these reasons, studies based on primary tumor biopsies, early-passage patient-derived xenografts, early-passage organoids that preserve the *in vivo* microenvironment, or malignant cells generated *de novo* directly from primary sources of normal human cells are anticipated to yield more convincing and informative data on cancer stem cell populations and their properties. However, as discussed below, their heterogeneity and finite availability also pose recognized challenges.

### Limitations Inherent in the Heterogeneity and Instability of Cancer Cell Populations

Conclusions based on generalizations and untested assumptions about the structure of cell populations within a tumor are often fraught with hazards. For example, cancer stem cell heterogeneity within a given tumor or class of tumors can be manifested in their other properties, and their identity can be confounded by the existence of progenitor cell populations with extensive proliferative capacity but not long term self-renewal potential, or the presence in some tumors of a majority cell population that is capable of both extensive long-term growth and self-renewal. These possibilities need to be taken into account in the experimental design and when deriving conclusions from the results obtained. Heterogeneity in tumor cell populations obtained from different patients (or other sources) also mandates that sufficient sample sizes be accrued and reported to support general conclusions.

### Take-Home Message

The field of cancer stem cells is an important one with great opportunities for the generation of new understanding of stem cell biology on a broad scale and clinical progress in the cancer field. It is also clear that cancer stem cell biology is a much more complex field than was envisaged when the concept was initially introduced decades ago. We thus remain very keen to receive and publish the wide range of studies that can inform any aspect of this topic in a significant and rigorous way without proscribing strict guidelines as to what these must include. We trust this Commentary will serve as a useful guide to the principles we seek to adhere to in evaluating the strength of research contributions to this exciting and dynamic area.
